# Emerging Roles of LSM Complexes in Posttranscriptional Regulation of Plant Response to Abiotic Stress

**DOI:** 10.3389/fpls.2019.00167

**Published:** 2019-02-19

**Authors:** Rafael Catalá, Cristian Carrasco-López, Carlos Perea-Resa, Tamara Hernández-Verdeja, Julio Salinas

**Affiliations:** Departamento de Biotecnología Microbiana y de Plantas, Centro de Investigaciones Biológicas-CSIC, Madrid, Spain

**Keywords:** LSM complexes, abiotic stress responses, Arabidopsis, posttranscriptional regulation, mRNA decapping, pre-mRNA splicing

## Abstract

It has long been assumed that the wide reprogramming of gene expression that modulates plant response to unfavorable environmental conditions is mainly controlled at the transcriptional level. A growing body of evidence, however, indicates that posttranscriptional regulatory mechanisms also play a relevant role in this control. Thus, the LSMs, a family of proteins involved in mRNA metabolism highly conserved in eukaryotes, have emerged as prominent regulators of plant tolerance to abiotic stress. Arabidopsis contains two main LSM ring-shaped heteroheptameric complexes, LSM1-7 and LSM2-8, with different subcellular localization and function. The LSM1-7 ring is part of the cytoplasmic decapping complex that regulates mRNA stability. On the other hand, the LSM2-8 complex accumulates in the nucleus to ensure appropriate levels of U6 snRNA and, therefore, correct pre-mRNA splicing. Recent studies reported unexpected results that led to a fundamental change in the assumed consideration that LSM complexes are mere components of the mRNA decapping and splicing cellular machineries. Indeed, these data have demonstrated that LSM1-7 and LSM2-8 rings operate in Arabidopsis by selecting specific RNA targets, depending on the environmental conditions. This specificity allows them to actively imposing particular gene expression patterns that fine-tune plant responses to abiotic stresses. In this review, we will summarize current and past knowledge on the role of LSM rings in modulating plant physiology, with special focus on their function in abiotic stress responses.

## Introduction

Due to their sessile nature, plants have evolved sophisticated adaptive mechanisms to correctly decipher external signals and deploy the corresponding adequate responses. This is of capital importance when facing adverse environmental conditions, since activating the right response can make the difference between life and death. In fact, abiotic stresses (i.e., extreme temperatures, drought or high salt concentration in the soil) are among the factors that most limit plant growth and development (Boyer, 1982; Bray et al., 2000). Plant responses to abiotic stresses, therefore, must be very precisely regulated. Results obtained in the last years have showed that these responses are mainly controlled by a wide reprogramming of gene expression (Seki et al., 2001; Kreps et al., 2002; Shinozaki et al., 2003). Several layers of regulation seem to be involved in shaping this reprogramming (Barrero-Gil and Salinas, 2013; Guerra et al., 2015). Among them, transcriptional control has attracted most of the attention so far and numerous transcription factors and *cis*-acting elements functioning in plant adaptation to abiotic stresses have been described (Khan et al., 2018). Nevertheless, different reports have pinpointed that posttranscriptional regulation also plays an essential role in modulating plant response to these challenging situations (Mazzucotelli et al., 2008; Floris et al., 2009; Nakaminami et al., 2012). In particular, the control of mRNA stability and precursor-mRNA (pre-mRNA) splicing, two crucial pathways of RNA metabolism, appear to fine tune plant adaptation to adverse environments.

**FIGURE 1:**
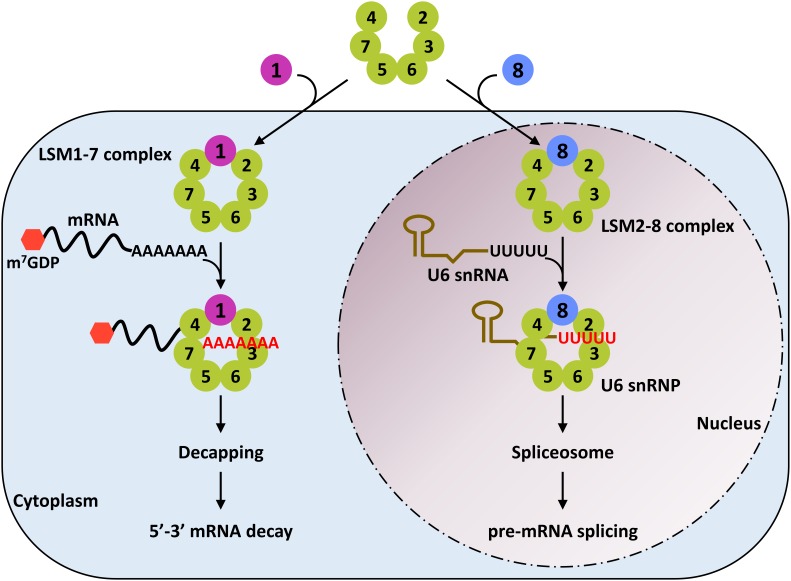
Subcellular localization and function of the eukaryotic LSM complexes. LSM1 protein promotes the assembly of the LSM1-7 complex in the cytoplasm. This complex is a critical component of the decapping machinery and, therefore, plays an essential role in the 5′-3′ mRNA decay pathway. LSM8, however, directs the formation of the LSM2-8 complex in the nucleus. This complex physically interacts with the oligo-U tract of the U6 snRNA to block its degradation by exonucleases. The LSM2-8 complex is a core component of the spliceosome and, coherently, participates in the splicing reaction.

The Sm-like proteins (LSMs) are implicated in numerous aspects of RNA metabolism in eukaryotes. The LSMs are evolutionary conserved RNA-binding proteins, typically arranged in two heteroheptameric ring-shaped complexes known as LSM1-7 and LSM2-8 (Figure 1) (Wilusz and Wilusz, 2013). The LSM1-7 ring is localized in the cytoplasm and is a structural component of the decapping machinery involved in exonucleolytic mRNA decay, while the LSM2-8 complex is localized in the nucleus and is a core component of the spliceosome (Wilusz and Wilusz, 2013). Using *Arabidopsis thaliana* (Arabidopsis) as a model plant, different investigations unveiled that LSM proteins are highly conserved in plants and that, as in other eukaryotes, are organized in cytoplasmic (LSM1-7) and nuclear (LSM2-8) complexes (Wang and Brendel, 2004; Cao et al., 2011; Perea-Resa et al., 2012; Golisz et al., 2013). Moreover, recent studies evidenced that both LSM rings actively participate in regulating plant responses to abiotic stress conditions (Perea-Resa et al., 2016; Carrasco-López et al., 2017), which constitutes an unanticipated novel function for the eukaryotic LSMs. In this review, we will summarize the current state of the art knowledge on the activity of LSM proteins, paying special attention to their role in modulating abiotic stress responses. First, in order to situate the LSM complexes into their own context, we will provide a general view about the RNA metabolic pathways in which they participate (i.e., the exonucleolytic mRNA decay and the pre-mRNA splicing), discussing the implication of their corresponding intermediates in plant response to abiotic stresses. Then, the function of LSM complexes in controlling plant adaptation to these adverse situations will be reviewed. Finally, we will propose and comment on future research directions to better understand the role of LSM complexes as master integrators of plant adaptation to their ever-changing environment.

## Posttranscriptional Regulation of Plant Response to Abiotic Stress

After transcription, mRNAs are subjected to different maturation and surveillance processes, which are indispensable to yield the functional transcripts. Differential control of the mechanisms implicated in these processes strongly influences not only the accumulation but also the structure of the final transcripts, significantly increasing the complexity of the information encoded by eukaryotic genomes (Schaefke et al., 2018). Plants may benefit from this layer of regulation since it provides a precise and reliable method to control gene expression, which, in turn, would ensure a timely response to environmental challenging situations. The LSM1-7 and LSM2-8 complexes are core components of two of the most influential posttranscriptional regulatory mechanisms namely, the mRNA decay and the pre-mRNA splicing processes, respectively. Before outlining the activity of the LSM complexes in mRNA decay and splicing, we will briefly describe the components of these two mechanisms, with special emphasis on their implication in plant response to abiotic stress.

### The Role of mRNA Decay Pathways in Plant Response to Abiotic Stress

The rate of mRNA decay ranges from minutes to several hours, depending on the transcripts (Chen and Coller, 2016). Control of mRNA decay provides a rapid instrument to regulate gene expression by modulating the stability of mRNAs. Two major pathways, the endonucleolytic and the exonucleolytic ones, govern transcript degradation (Garneau et al., 2007). The endonucleolytic cleavage pathway includes quality control mechanisms, such as the nonsense-mediated decay (NMD), that functions to prevent translation of error-containing mRNAs, or the posttranscriptional gene silencing (PTGS), mainly involved in the control of gene expression (Almeida and Allshire, 2005; Nasif et al., 2018). On the other hand, the exonucleolytic pathway is characteristic of transcripts that have performed their function and are no longer needed (Kilchert et al., 2016). This route starts with the shortening of the poly(A) tail positioned at the 3′ end of the mRNAs, a process named deadenylation (Abbasi et al., 2013). Subsequently, transcripts can be degraded in a 3′-5′ direction by the exosome or the exoribonuclease SUPPRESSOR OF VCS (SOV)/DIS3L2 (Abbasi et al., 2013; Soma et al., 2017). Alternatively, mRNAs can lose their 5′ N7-methylguanosine (m^7^GDP) cap (5′ CAP), by the action of the decapping complex, and then be degraded in 5′-3′ direction through 5′-3′ exoribonucleases (XRNs) (Jonas and Izaurralde, 2013).

#### mRNA Deadenylation

**FIGURE 2:**
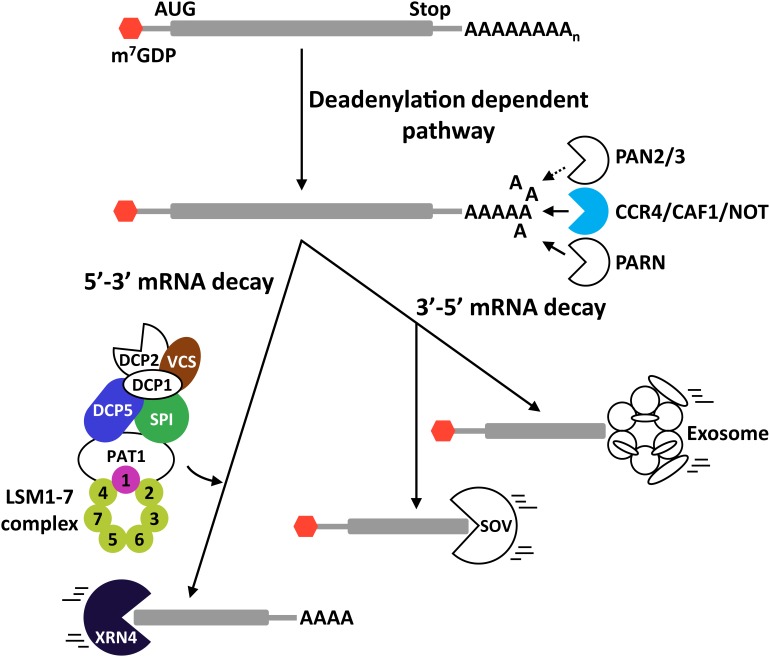
Exonucleolytic mRNA degradation pathways in plants. mRNA deadenylation can be performed through three different pathways mediated by the PAN2/3 complex, the CCR4/CAF1/NOT complex, or the PARN enzyme. After deadenylation, mRNAs may be degraded in 3′-5′ direction (3′-5′ mRNA decay) or in 5′-3′ direction (5′-3′mRNA decay). In the 3′-5′ route, transcripts are degraded by the exosome or by SOV. In the case of the 5′-3′ route, it is imperative to remove the 5′ CAP structure of the transcripts by the decapping complex before their degradation by XRN4. Proteins in color represent proteins that participate in regulating plant tolerance to abiotic stress. DCP5 and VCS are positive regulators of plant tolerance to drought. SPI positively regulates Arabidopsis tolerance to high salt. The LSM1-7 complex attenuates tolerance to drought and the cold acclimation process, while promoting salt stress tolerance. Solid and dotted arrows represent established and theoretical pathways, respectively.

In eukaryotes, three major pathways control the deadenylation of poly(A) RNAs. They are defined by the participation of the PAN2-PAN3 (PAN2/3) complex, the Carbon Catabolite Repressor 4 (CCR4)/CCR4-Associated Factor 1 (CAF1)/NOT (CCR4/CAF1/NOT) complex, or the poly(A) ribonuclease PARN (Abbasi et al., 2013) (Figure 2). While PAN2/3 complex activity has not yet been reported in plants, recent data have revealed the implication of the two other pathways in different plant physiological processes. Nevertheless, data demonstrating their role in plant adaptation to abiotic stress remain scarce. Arabidopsis has a functional poly(A) ribonuclease, known as AtPARN, that mediates the deadenylation of transcripts induced during embryo development (Chiba et al., 2004; Reverdatto et al., 2004). The induction of *AtPARN* transcription by osmotic and salt stresses (Nishimura et al., 2005) suggests a possible function of this ribonuclease in plant tolerance to these challenging situations, although no data has been reported in this regard. In yeast, the CCR4/CAF1/NOT complex consists of a catalytic center composed by three DEDD-type nucleases (CAF1, CAF40, CAF130) and one EEP-type endonuclease (CCR4), and five non-catalytic NOT proteins (NOT1 to NOT5) (Goldstrohm and Wickens, 2008; Abbasi et al., 2013). Plant homologs to NOT proteins have not yet been reported. Suzuki et al. (2015), however, demonstrated that Arabidopsis has two functional CCR4 proteins, AtCCR4a and AtCCR4b, which have been described to control the deadenylation of starch-biosynthesis-related genes and, coherently, the starch levels. Whether AtCCR4a and AtCCR4b are involved in plant response to abiotic stress remains to be determined. On the other hand, Arabidopsis contains 11 genes encoding proteins with high identity to CAF1 (Walley et al., 2010), and two of them, AtCAF1a and AtCAF1b, display deadenylation activity (Liang et al., 2009). Interestingly, both proteins are implicated in the photoxidative stress response, and, furthermore, AtCAF1a in plant tolerance to salt stress controlling mRNA decay (Walley et al., 2010).

#### The 3′-5′ Degradation Pathway

After poly(A) shortening by deadenylases, transcripts can be degraded in 3′-5′ direction through the exosome (Chlebowski et al., 2013) or the SOV pathways (Zhang et al., 2010; Sorenson et al., 2018) (Figure 2). The eukaryotic exosome is a highly conserved macromolecular complex whose core is composed by nine proteins that are accompanied by different accessory proteins for target recognition, such as RNA-binding proteins and RNA helicases (Chlebowski et al., 2013; Thoms et al., 2015). The plant exosome has been involved in the control of different physiological processes, including cuticular wax biosynthesis (Hooker et al., 2007), seed germination and early seedling growth (Yang et al., 2013), and female gametogenesis and embryo development (Chekanova et al., 2007). Whether it operates in plant response to abiotic stress conditions remains to be assessed. SOV is a 3′-5′ exoribonuclease that was identified as a suppressor of null mutations in *VARICOSE* (*VCS*), a key component of the decapping complex (see below) (Zhang et al., 2010). Recent discoveries indicated that SOV and VCS would control the decay of numerous transcripts through an elaborated feedback regulatory system (Sorenson et al., 2018). Null mutations in *SOV* do not produce any significant morphological phenotype, but their tolerance to abiotic stress has not been studied and, thus, a possible implication cannot be ruled out.

#### The 5′-3′ Degradation Pathway

Following deadenylation, transcripts can also be degraded in 5′-3′ direction by the action of XRNs (Figure 2). This pathway controls the decay of around 68% of all transcripts in Arabidopsis and, thus, is considered the most influential mRNA decay system in plants (Sorenson et al., 2018). Prior to the action of the XRNs, the 5′ CAP that protects the 5′ end of mRNAs from degradation needs to be removed by the decapping complex. During this process, the decapping complex generates mRNAs with a monophosphate nucleotide in their 5′ end that are the preferential substrates of XRNs (Nagarajan et al., 2013; Kurihara, 2017). The decapping reaction and the subsequent mRNA degradation occurs in discreet cytoplasmic foci known as processing bodies (P-bodies), where the target mRNAs and the degradation factors assemble (Sheth and Parker, 2003; Maldonado-Bonilla, 2014). The components of eukaryotic decapping machinery can be divided into two groups depending on their function. The first group contains the holoenzyme formed by DCP2 and DCP1. DCP2 has specific pyrophosphatase activity for removing the 5′ CAP, and DCP1 functions as an activator of DCP2 (Coller and Parker, 2004). The second group is composed by different regulatory proteins that are required for efficient decapping, including the Protein Associated with Topoisomerase II (PAT1), the DEAD box helicase Dhh1p, and the RNA-binding complex LSM1-7 (Coller and Parker, 2004). Xu et al. (2006) showed that Arabidopsis has DCP2 and DCP1 proteins with similar roles as those of other eukaryotes, and that VCS, a previously described Arabidopsis protein (Deyholos et al., 2003), is the functional homolog of the human DCP2 activator Hedls/Ge-1. DCP5, in turn, was demonstrated to be the Arabidopsis homolog of the Dhh1p protein (Xu and Chua, 2009). The Arabidopsis protein DGP SPIRRING (SPI), furthermore, has been described to interact with DCP1 and, consequently, to be part of the decapping complex (Steffens et al., 2015) (Figure 2).

Different studies have involved the decapping complex in plant response to abiotic stress. For example, DCP2 and DCP1 accumulate to P-bodies in response to heat stress and low temperature, respectively (Perea-Resa et al., 2016). Nonetheless, these studies did not provide experimental evidence on the implication of these proteins in plant tolerance to abiotic stress. Interestingly, when plants are exposed to osmotic stress, the phosphorylation of DCP1 by the MAP protein kinase 6 (MPK6) promotes its interaction with DCP5 to control the decay rate of stress-related transcripts and, consequently, Arabidopsis tolerance to this adverse situation (Xu and Chua, 2012). SPI positively controls plant response to salt stress mediating the proper localization of salt stress-related transcripts at the P-bodies (Steffens et al., 2015). Whether this function is mediated through its interaction with DCP1 remains unknown. VCS has been recently reported as a SnRK2s substrate when plants are exposed to osmotic stress (Soma et al., 2017). The phosphorylation of VCS by SRK2G/SnRK2.1 is required, under osmotic stress, for the adequate mRNA decay of transcripts encoding regulators of plant tolerance to drought (Soma et al., 2017). Finally, it has been well documented that Arabidopsis has a functional LSM1-7 complex that plays important roles in the control of plant adaptation to several abiotic stresses by governing the decay rate of transcripts corresponding to key regulators of plant responses to those conditions (Perea-Resa et al., 2012; Golisz et al., 2013; Perea-Resa et al., 2016; Wawer et al., 2018).

After the 5′ mRNA decapping, XRN proteins degrade transcripts (Figure 2). The XRN family is highly conserved in eukaryotes and is mainly characterized by the existence of one or more nuclear enzymes (XRN2/RAT1 and XRN3), and one cytoplasmic enzyme (XRN1/PACMAN or XRN4) (Nagarajan et al., 2013). In Arabidopsis, there is not a homologous protein to XRN1 but there are three proteins with high identity to XRN2 (AtXRN2, AtXRN3, and AtXRN4), at both structural and functional levels (Kastenmayer et al., 2000). AtXRN2 and AtXRN3 are localized in the nucleus while AtXRN4 is localized in the cytoplasm (Kastenmayer et al., 2000). Only AtXRN4 has been related with plant tolerance to abiotic stresses, in particular to heat stress (Merret et al., 2013; Nguyen et al., 2015). Recent data have also implicated AtXRN4 in Arabidopsis sensitivity to ABA, suggesting that it could play a more general function in abiotic stress responses (Wawer et al., 2018).

### The Role of Pre-mRNA Splicing in Plant Response to Abiotic Stress

Introns were identified in the late 1970’s as non-coding DNA sequences interrupting the coding sequence of adenovirus genes (Berget et al., 1977; Chow et al., 1977). Nowadays, it is known that introns are not exclusive of adenovirus but are widely present in all eukaryotic genomes (Matera and Wang, 2014).

#### The Core of the Spliceosome

Lerner et al. (1980) proposed that introns were spliced from the pre-mRNAs by a highly dynamic association of five small nuclear ribonucleoproteins (snRNPs), namely U1, U2, U4, U5, and U6, in a higher order complex known as spliceosome. Further investigations confirmed this assumption and revealed that these five snRNPs are associated with more than 300 proteins that coordinately participate in the control of spliceosome activity (Wahl et al., 2009; Matera and Wang, 2014). snRNPs are evolutionary conserved and their core is composed of a small uridine-rich nuclear RNA (snRNA), which defines the complex (i.e., U1, U2, U4, U5, and U6 snRNAs), and an accompanying heteroheptameric protein complex (Wilusz and Wilusz, 2013). Depending on the complex, the snRNPs can be divided in two groups. The first one, which has the Sm complex as protein moiety, includes the U1, U2, U4, and U5 snRNPs. The second group is formed by the U6 snRNP, which is accompanied by the LSM2-8 complex (Wilusz and Wilusz, 2013). The main function of the Sm complex is to guarantee the correct levels of U1, U2, U4, and U5 snRNAs (Will and Lührmann, 2011). The LSM2-8 complex participates in the biogenesis of the U6 snRNA, ensuring its stability and adequate levels (Wilusz and Wilusz, 2013). Recent reports revealed that plants genomes encode proteins for all components of the Sm and LSM2-8 complexes (Wang and Brendel, 2004; Cao et al., 2011; Perea-Resa et al., 2012; Golisz et al., 2013). The characterization of the Arabidopsis LSM2-8 ring not only confirmed that plants have a functional LSM2-8 ring but also that it displays unexpected functions in controlling the spliceosome activity (Perea-Resa et al., 2012; Carrasco-López et al., 2017) (see below).

#### The Splicing Reaction

**FIGURE 3:**
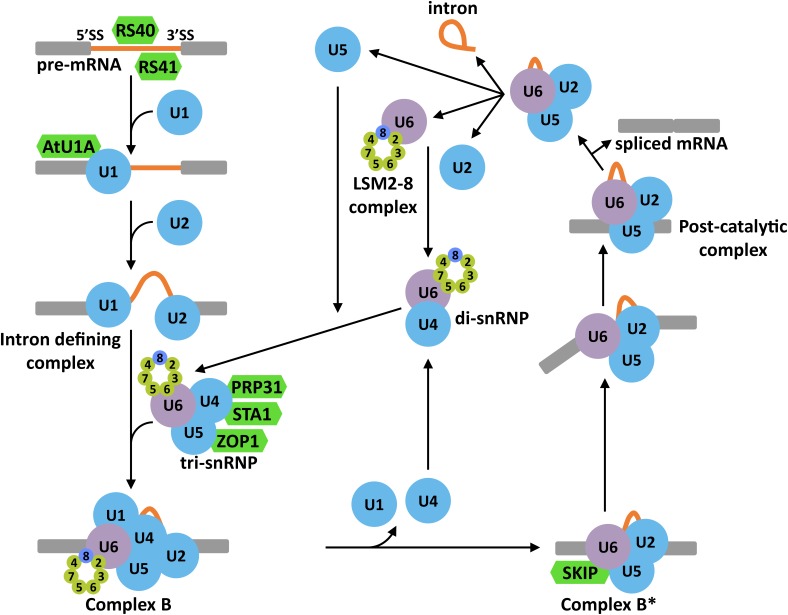
Schematic representation of the splicing reaction in eukaryotes. 5′ and 3′ splice sites (SS) are recognized by SR proteins that attract the U1 and U2 snRNPs to constitute the intron defining complex. Then, the U4/U6.U5 tri-snRNP is recruited to the intron region to form the complex B. The exclusion of U1 and U4 snRNPs would rise to the complex B^∗^ which, together with several RNA helicases, is responsible of the first and second catalytic reactions. These two reactions render the post-catalytic complex that contains the intron lariat, the ligated exons and the U4/U6.U5 tri-snRNP. The release of the snRNPs from this complex allows the regeneration of the spliceosome for subsequent splicing reactions. In green color are represented Arabidopsis proteins with known functions in plant tolerance to abiotic stress.

In the model of the splicing reaction accepted so far, intron scission from a pre-mRNA implies several heavily regulated steps (Figure 3) (review in Matera and Wang, 2014). The first one is the choice of the splice site (SS). This step is mediated by different *cis*-acting elements present in the sequence of the pre-mRNA, which are recognized by *trans*-acting factors (i.e., Serine/arginine-rich protein (SR) or heterogeneous nuclear ribonucleoproteins (hnRNPs)). These factors seem to be the ultimate responsible for attracting the first components of the spliceosome, the U1 and U2 snRNPs. The U1 snRNP binds to the 5′ SS, and the U2 snRNP, with associated factors, to the 3′ SS and the branch point to define the so called intron defining complex. After the creation of this complex, the U4/U6.U5 tri-snRNP is recruited to give rise to the precatalytic complex (complex B). Then, the complex B is activated (complex B^∗^) by the action of different RNA helicases, resulting in the liberation of the U1 and U4 snRNPs and, more relevant, in the first catalytic step that renders a free 5′ exon and an intron-3′ exon lariat intermediate. The second catalytic step is mediated by the action of several RNA helicases and ends up with the generation of the post-catalytic complex containing the intron lariat and the joined exons. After this second reaction, all snRNPs are released so they can follow a subsequent round of splicing.

#### Alternative Splicing in Plant Response to Abiotic Stress

The alternative selection of SS, a process known as alternative splicing (AS), is emerging as one of the most versatile regulatory systems in eukaryotes. By means of this process, different functional mRNAs can be generated from a particular pre-mRNA through different arrangements of introns and exons, significantly increasing the protein diversity (Black, 2003; Nilsen and Graveley, 2010). Moreover, the AS can also participate in controlling the levels of functional mRNAs because of the generation of error-containing transcripts that are targets of mRNA surveillance pathways like the NMD (Staiger and Brown, 2013). In plants, AS has a pivotal role in the regulation of gene expression affecting about 60% of all intron-containing genes (Zhang et al., 2017). In response to abiotic stresses, AS seems to have a particular important function since several key regulatory genes of plant tolerance to such stresses have been shown to be prone to AS events (Deng et al., 2011; Seo et al., 2012, 2013; Leviatan et al., 2013; Liu et al., 2013; Ding et al., 2014a; Calixto et al., 2018). Ding et al. (2014a) reported that around 49% of all intron-containing genes in Arabidopsis are subjected to AS in response to salt stress. Similarly, when plants are exposed to low temperature, more than 2000 genes display changes in their splicing patterns, including some important regulators of cold tolerance (i.e., PHYB or PIF7) (Calixto et al., 2018). Interestingly, among the 2000 genes showing differential AS when plants are exposed to low temperature, some are well known splicing factors, such as RCF1, GEMIN2, or STA1 (Calixto et al., 2018), indicating that cold also modulates the spliceosome activity. Accordingly, several splicing factors have been reported to play key functions in plant response to abiotic stress (extensively revised in Laloum et al., 2017; Calixto et al., 2018) (Figure 3). Thus, Arabidopsis RS40 and RS41, two SR proteins likely involved in SS recognition, are required for the correct splicing of several pre-mRNAs under high salt conditions and, moreover, for plant tolerance to this adverse situation (Chen et al., 2013). Similarly, AtU1A, the Arabidopsis homolog of human U1A, a component of the U1 snRNP, promotes salt tolerance and determines the 5′ SS selection, shaping the splicing patterns in response to high salt (Gu et al., 2018). PRP31, the Arabidopsis homolog of a subunit of the U4/U6.U5 tri-snRNP in human and yeast, has been demonstrated to ensure the correct splicing of a number of pre-mRNAs under high salt, mannitol and low temperature and plant tolerance to these abiotic stresses (Du et al., 2015). Other components of this complex, such as STA1 and ZOP1, have also been implicated in controlling pre-mRNA splicing and tolerance to abiotic adverse conditions, including low temperature, high salt and osmotic stress (Lee et al., 2006; Du et al., 2015). Furthermore, SKIP, a component of the Arabidopsis complex B^∗^ (Li et al., 2016), has been shown to regulate plant tolerance to high salt by determining the correct splicing pattern under this challenging environment (Feng et al., 2015).

## The Eukaryotic Lsm Proteins

The eukaryotic LSM proteins belong to the large family of “Sm-like” proteins (Tharun, 2008). They were identified as antigens recognized by antibodies of systemic lupus erythematosus, and were named after the patient that provided the serum [i.e., Stephanie Smith (Sm)] (Tan and Kunkel, 1966). Later on, Lerner et al. (1981) reported for the first time that LSM proteins are complexed with different snRNAs to form snRNPs. They are evolutionary conserved from Archaebacteria and prokaryotes to eukaryotes, indicating that they could be already present in the last universal common ancestor (LUCA) (Anantharaman et al., 2002). Typically, the LSM proteins are small peptides (∼10–25 kDa) with common structural features. Their sequences contain a highly conserved bipartite domain, the Sm-domain, spanning over 100 conserved residues interrupted by a non-conserved region of up to 30 amino acids (Hermann et al., 1995; Séraphin, 1995; Achsel et al., 1999; Kambach et al., 1999; Salgado-Garrido et al., 1999). The tertiary structure displays a characteristic fold containing an N-terminal helix adjacent to a strongly bent five-stranded antiparallel β-sheet, the so-called “Sm-fold” (Kambach et al., 1999). The LSM proteins tend to arrange in ring shaped hetero-heptameric complexes with RNA-binding capability (Raker et al., 1996; Mayes et al., 1999; Salgado-Garrido et al., 1999). These rings ensure the correct levels of U6 snRNAs and facilitate the assembly of snRNP complexes (Tharun, 2008; Wilusz and Wilusz, 2013).

The LSM protein family can be divided into the Sm and the LSM subfamilies. The Sm subfamily is mainly composed by seven proteins (SmB/B′, SmD1, SmD2, SmD3, SmE, SmF, and SmG) assembled in heteroheptameric complexes around the snRNAs of the major (U1, U2, U4, and U5 snRNAs) and minor (U11, U12, and U4atac snRNAs) spliceosomes (Hermann et al., 1995; Will et al., 1999). On the other hand, the LSM subfamily is composed of eight proteins (LSM1-LSM8) that are also organized in heteroheptameric complexes (Zhou et al., 2014). Six of them are homologous to the Sms (LSM2, LSM3, LSM4, LSM5, LSM6, and LSM7), LSM8 is weakly related to SmB/B′, and LSM1 does not display significant similarity to any Sm protein (Salgado-Garrido et al., 1999). LSM1 and LSM8 proteins are mutually exclusive and determine the subcellular localization of the complexes and, more relevant, their function (Salgado-Garrido et al., 1999; Tharun et al., 2000) (Figure 1). LSM1 promotes the constitution of an LSM1-7 complex in the cytoplasm that plays a crucial role in the decapping complex (Salgado-Garrido et al., 1999). In contrast, LSM8 directs the formation of the LSM2-8 complex that localizes in the nucleus and forms, together with the U6 snRNA, the U6 snRNP (Séraphin, 1995; Salgado-Garrido et al., 1999). Both LSM complexes share preference for the 3′ ends of RNAs, the LSM1-7 ring for 3′ oligoadenylated tracts, and the LSM2-8 for 3′ oligouridinylated (oligo-U) tracts (Chowdhury et al., 2007; Zhou et al., 2014). In addition to the canonical LSMs, there are at least three classes of larger proteins having Sm-domains and other functional domains with RNA-binding capability. These LSM proteins are not well characterized, although it seems that some of them are related to mRNA translational control (Decker and Parker, 2006).

### The LSM1-7 Complex

**FIGURE 4:**
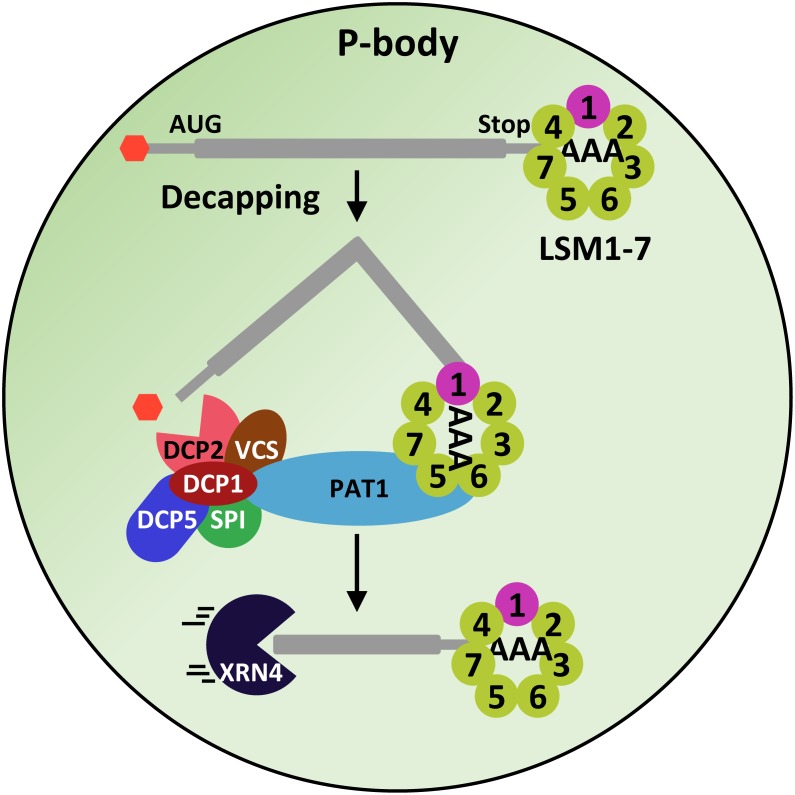
The LSM1-7 complex participates in the decapping machinery. The LSM1-7 complex would interact with the oligo-A tract of mRNAs and through its interaction with the scaffold protein PAT1, it would recruit the DCP1, DCP2, VCS, DCP5 and SPI proteins, which, in turn, would remove the 5′CAP of mRNAs, allowing its subsequent 5′-3′ degradation by XRN4.

When the eukaryotic LSM proteins were identified, it soon became clear that one of them, the LSM1, did not bind to the U6 snRNA (Salgado-Garrido et al., 1999). Furthermore, the deletion of LSM1 neither affected the levels of U6 snRNA nor the splicing efficiency (Mayes et al., 1999; Salgado-Garrido et al., 1999), indicating that LSM1 has a different function than the LSM2-8 complex. Indeed, it is a component of the 5′-3′ mRNA degradation pathway mediating the decapping of several mRNAs (Boeck et al., 1998) (Figure 1). On the other hand, further analyses revealed that, in yeast, the proteins LSM2 to LSM7 participate in the control of mRNA decapping, while LSM8 does not (Tharun et al., 2000). Consistent with these results, LSM1 to LSM7 proteins co-localize and co-immunoprecipitate with DCP1, PAT1 and different mRNAs (Bouveret et al., 2000; Tharun et al., 2000). Similarly, the human LSM1-7 complex associates with PAT1 and XRN1 to modulate mRNA degradation (Ingelfinger et al., 2002). It was proposed that LSM1-7 proteins are arranged in a complex different than that of the LSM2-8 nuclear one, which would be a component of the decapping complex (Tharun et al., 2000). This hypothesis was validated by crystallization experiments, which demonstrated that the yeast LSM1-7 complex resembles a thick donut, generated by the sequential interaction of the seven proteins (LSM1-LSM2-LSM3-LSM6-LSM5-LSM7-LSM4) through their Sm domains (Sharif and Conti, 2013) (Figure 1). The ring organization of the complex yields a lumen where the spacing between subunits matches the space between nucleotides in the RNA, allowing its interaction with single-stranded RNAs (Khusial et al., 2005). The LSM1-7 complex holds high affinity for mRNAs containing oligo-A tracts of 6 or more nucleotides (oligoadenilated) at its 3′ terminal end (Chowdhury et al., 2007). In the model proposed for its function in regulating the decapping reaction, the complex would bind to the 3′ end of the oligoadenylated mRNAs in P-bodies (Chowdhury et al., 2007; Tharun, 2008) (Figure 4).

#### The LSM1-7 Complex in Plants

A bioinformatic approach allowed the first report about plant LSM proteins. Séraphin (1995) found that the genomes of alfalfa, Arabidopsis, *Brassica campestris* and rice contained genes encoding proteins with high sequence identity to the Sm proteins of yeast and animals. The Arabidopsis genome, in particular, has been described to contain around 42 genes encoding proteins with the characteristic Sm domain (Wang and Brendel, 2004; Cao et al., 2011; Perea-Resa et al., 2012; Golisz et al., 2013). Eleven Arabidopsis genes encode the eight canonical LSM proteins, LSM1-LSM8, from yeast and animals (Perea-Resa et al., 2012; Golisz et al., 2013). Genes *LSM1*, *LSM3*, and *LSM6* are duplicated and encode pairs of redundant proteins (LSM1A, B; LSM3A, B and LSM6A, B). As in yeast and animals, Arabidopsis LSM proteins are arranged in two main complexes, the cytoplasmic LSM1-7 and the nuclear LSM2-8 ones (Perea-Resa et al., 2012; Golisz et al., 2013). Fortunately, Arabidopsis null mutants *lsm1alsm1b* and *lsm8* resulted to be viable, providing unique genetic tools to approach the functional characterization of the two LSM complexes (Perea-Resa et al., 2012; Golisz et al., 2013).

The functional characterization of *lsm1alsm1b* double mutants revealed that, as in other eukaryotes, Arabidopsis LSM1 proteins are essential for the constitution of the LSM1-7 cytoplasmic ring (Perea-Resa et al., 2012). The Arabidopsis LSM1-7 complex co-immunoprecipitate with PAT1 and, moreover, co-localize with DCP2 and VCS in P-bodies, strongly suggesting that, together with DCP1, DCP5 and SPI, it participates in the Arabidopsis decapping machinery (Perea-Resa et al., 2012; Golisz et al., 2013) (Figure 4). Indeed, it has been shown to be essential for the correct decapping of a plethora of selected mRNAs, ensuring their precise turnover (Perea-Resa et al., 2012; Golisz et al., 2013). Interestingly, among the LSM1-7 targets, several transcripts corresponding to genes having key roles in Arabidopsis development were found (Perea-Resa et al., 2012; Golisz et al., 2013), which would account for the developmental alterations displayed by the *lsm1alsm1b* plants (Perea-Resa et al., 2012; Golisz et al., 2013). All these data highlight the relevance of the LSM1-7 complex in shaping plant physiology by modulating mRNA turnover.

#### The LSM1-7 Complex in Plant Response to Abiotic Stress

**FIGURE 5:**
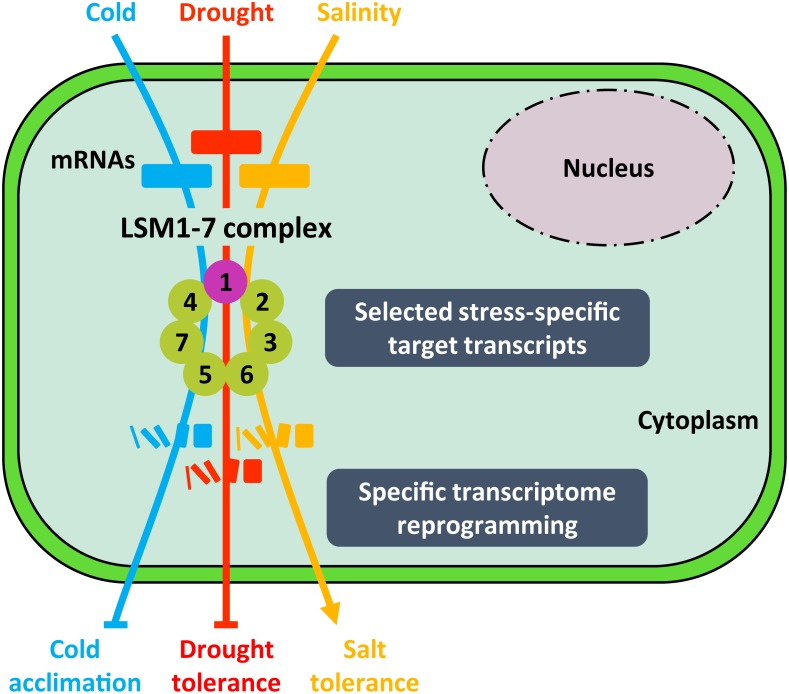
Proposed model for the function of LSM1-7 complex in plant response to abiotic stresses. Depending on the adverse environmental condition (i.e., low temperature, drought or high salinity), the LSM1-7 complex would target stress-specific transcripts encoding essential regulators of plant tolerance to the corresponding stress situation, promoting their decay and, thus, giving rise to an specific gene expression profile. Arrowheads and end lines indicate positive and negative regulation, respectively.

Genetic and molecular evidence unveiled that the LSM1-7 complex has a pivotal role in regulating plant response to abiotic stress. The expression levels of *LSM1* significantly increase when plants are exposed to low temperature and, accordingly, the levels of the corresponding protein augment as well (Perea-Resa et al., 2016). In contrast, the levels of *LSM1* transcripts do not change in response to other abiotic stresses, such as high temperature, high salt or drought (Okamoto et al., 2016; Perea-Resa et al., 2016). The functional characterization of Arabidopsis LSM5 and LSM4 proteins showed that they are implicated in plant response to abiotic stress (Xiong et al., 2001; Zhang et al., 2011). A point-mutation allele of *LSM5, sad1*, displayed reduced tolerance to salt and drought stresses compared to wild-type plants (Xiong et al., 2001). Similarly, a null mutant for *LSM4* showed decreased salt stress tolerance (Zhang et al., 2011). Although the implication of these two proteins in the decapping reaction under abiotic stress was not evaluated, it was later demonstrated that LSM5/SAD1 promotes mRNA degradation under control conditions by removing the 5′CAP in Arabidopsis (Golisz et al., 2013). Nonetheless, taken into account that LSM4 and LSM5/SAD1 are shared components of the cytoplasmic and nuclear LSM complexes, it is difficult to discriminate if their function in abiotic stress response is mediated through the LSM1-7 complex, the LSM2-8 complex or both. The characterization of the *lsm1alsm1b* double mutant, however, provided definitive evidence that the LSM1-7 complex is necessary for the correct adaptation of plants to situations of abiotic stress. It negatively regulates the ability of Arabidopsis to cold acclimate and tolerate drought, but functions as a positive regulator of Arabidopsis tolerance to salt stress (Perea-Resa et al., 2016). Genome-wide expression analysis of *lsm1alsm1b* plants unveiled that the LSM cytoplasmic complex differentially regulates Arabidopsis response to abiotic stresses by differentially controlling the levels of stress-inducible transcripts depending on the stress (Perea-Resa et al., 2016). Additional characterization of *lsm1alsm1b* plants unveiled an unexpected functional plasticity of the LSM1-7 complex to modulate the interaction of plants with their environment. In fact, depending on the abiotic stress conditions, the complex interacts with selected stress-inducible transcripts, such as *LEA7*, *ZAT12*, *ABR1*, *ANAC019*, *AHK5*, or *ANAC092*, targeting them for decapping and subsequent degradation, ensuring the appropriate patterns of downstream stress-responsive gene expression that are required for plant adaptation (Perea-Resa et al., 2016) (Figure 5). This stress-dependent differential control of mRNA turnover represents a new layer of regulation in plant adaptation to unfavorable environmental conditions. Remarkably, it was demonstrated that the LSM cytoplasmic complex is required for the constitution of P-bodies in plants under abiotic stress conditions (Perea-Resa et al., 2016). Furthermore, the exposure of Arabidopsis plants to these conditions promotes the accumulation of the LSM1-7 complex in P-bodies (Perea-Resa et al., 2016). Given that other P-body constituents such as DCP1 and VCS have also been shown to accumulate there in response to abiotic stresses (Perea-Resa et al., 2016), it seems reasonable to hypothesize that all components of the decapping machinery concentrate in these cytoplasmic foci when plants are confronted to adverse environmental conditions to govern mRNA decay.

#### The Role of LSM1-7 Complex in the Control of Stress-Induced ABA Biosynthesis

The increase of abscisic acid (ABA) levels is one of the primary signals triggering adaptive responses when plants are exposed to abiotic stresses such as low temperature, drought or high salt (Yang et al., 2017). ABA biosynthesis is tightly regulated at different levels, the posttranscriptional one being among the most relevant (Yang et al., 2017). Xiong et al. (2001) evidenced the pivotal role of LSM proteins in regulating ABA biosynthesis and signaling. The characterization of mutant plants with altered function of LSM5/SAD1 revealed that this LSM subunit controls the levels of transcripts corresponding to key intermediates in ABA biosynthesis (i.e., AAO3 or ABA3) and signaling (i.e., PP2C) (Xiong et al., 2001). Nonetheless, as already mentioned, the participation of LSM5/SAD1 in LSM1-7 and LSM2-8 complexes prevent to determine the actual mechanisms through which such control is carried out. Recent analyses of the *lsm1alsm1b* mutant have shed some light to this conundrum. Perea-Resa et al. (2016) reported that *lsm1alsm1b* displays increased levels of ABA in response to low temperature and high salt, but not in response to water stress, indicating that the LSM1-7 complex differentially regulates stress-induced ABA biosynthesis. They demonstrated that, depending on the stress situation, the LSM ring exerts this function by differentially controlling the decapping of *NCED3* and *NCED5* mRNAs, two transcripts encoding key ABA biosynthetic enzymes, and, therefore, their decay rate. The LSM1-7 complex attenuates ABA biosynthesis under cold conditions by interacting with *NCED3* and *NCED5* mRNAs, and under salt stress with *NCED5* mRNA, promoting their degradation. None of them, however, is target of the complex in response to water stress (Perea-Resa et al., 2016). On the other hand, it has been proposed that ABA perception and signaling through the canonical PYL/PYR/RCAR-PP2C-SnRK2 pathway is governed by the decapping complex (Wawer et al., 2018). Thus, the decay rate of the mRNAs encoding the ABA receptor PYR1 and the ABA-unresponsive SnRK2 protein kinases would be determined by the LSM1-7 complex, DCP5, and XRN4 (Wawer et al., 2018). Still, whether this activity of the decapping machinery is also involved in plant adaptation to abiotic stress conditions remains to be investigated. All these results provide genetic and molecular evidence that the LSM cytoplasmic complex contributes to establish the appropriate levels of ABA in Arabidopsis plants exposed to different abiotic stresses.

### The LSM2-8 Complex

**FIGURE 6:**
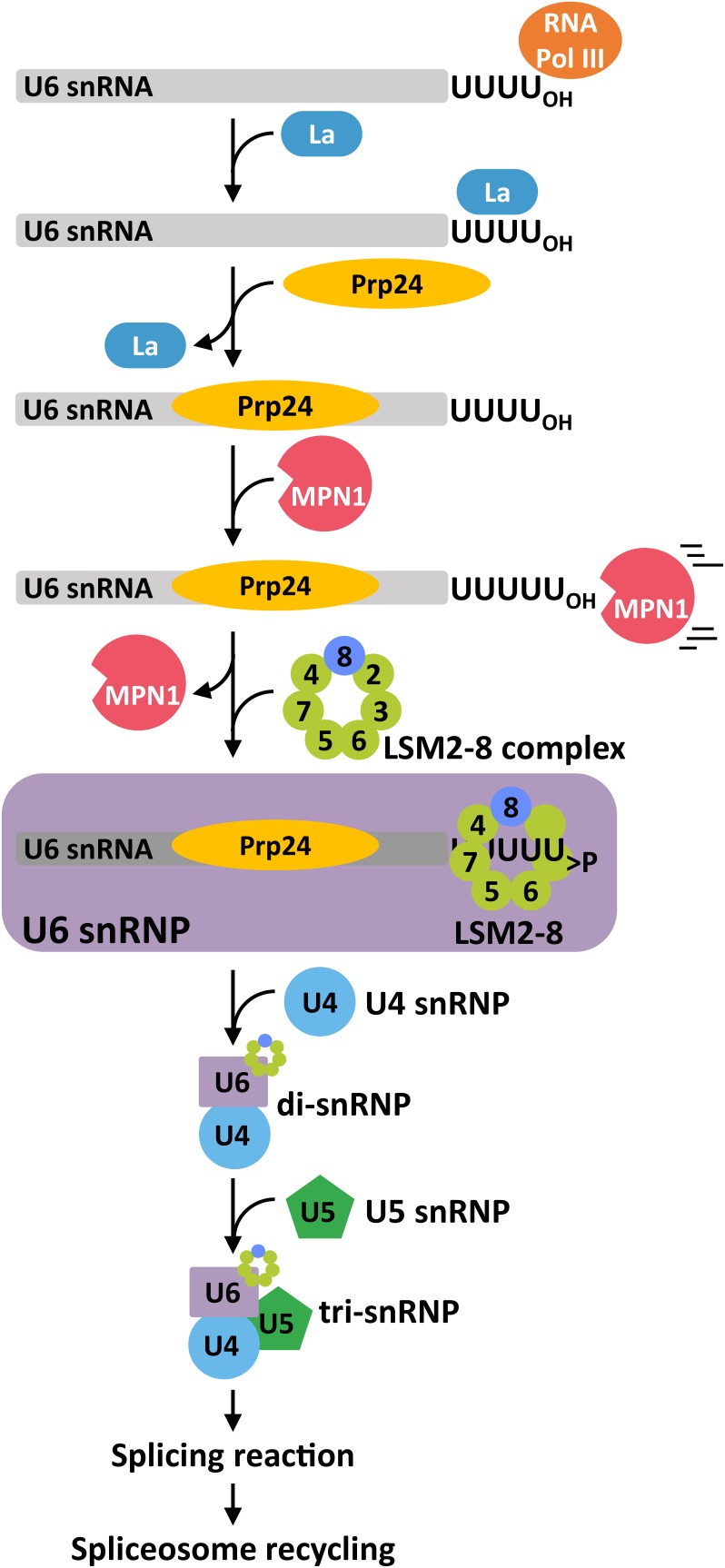
Representative model for the function of LSM2-8 complex in U6 snRNA maturation, formation of the di- and tri-snRNPs, and splicesome regeneration. After its transcription by the Pol III, the U6 snRNA is stabilized by the binding of the La protein to its oligo-U tract. The binding to the body of the snRNA of Prp24 and the excision of the last U by MPN1/USB1/UBL1 (MPN1) promote the substitution of MPN1 by the LSM2-8 complex. The U6 snRNA, Prp24 and the LSM2-8 complex constitute the U6 snRNP that, subsequently, favors the formation of U4/U6 di- and U4/U6.U5 tri-snRNPs. In addition, after the splicing reaction a fully functional LSM2-8 complex is essential for the regeneration of the spliceosome.

As already mentioned, the eukaryotic LSM2-8 complex was initially identified in the nucleus, associated to the U6 snRNA (Salgado-Garrido et al., 1999) (Figure 1). The complex, composed by proteins LSM2 to LSM8 sequentially ordered (LSM2-LSM3-LSM6-LSM5-LSM7-LSM4-LSM8) in a ring shape (Zhou et al., 2014), displays preference for oligo-U tracts (Achsel et al., 1999; Mayes et al., 1999), a typical feature of RNAs transcribed by the RNA polymerase III (Pol III). It has been described that the LSM nuclear complex participates in different crucial processes of pre-mRNA splicing, such as the biogenesis of the U6 snRNA, the constitution of U4/U6 di-snRNP and U4/U6.U5 tri-snRNP complexes, or the regeneration of the spliceosome (reviewed in Didychuk et al., 2018) (Figure 6). The U6 snRNA is part of the catalytic core of the spliceosome and, thus, its levels should be subjected to a tight control. Just after its transcription by the RNA Pol III, the chaperone-like La/Lhp1 protein interacts with the U6 snRNA Poly(U) tract to promote its retention in the nucleus. This interaction is weakened by the binding of the RNA-binding protein Prp24, allowing the access of the 3′ to 5′ RNA exonuclease MPN1/USB1/UBL1 that removes the last uridine moiety and leaves a phosphate in the 3′ end of the transcript. Then, the presence of this phosphate favors the interaction of the LSM2-8 complex with the 3′ end of the U6 snRNA. The binding of this complex inhibits U6 snRNA degradation, retains the transcript in the nucleus, and is required for the proper formation of the U6 snRNP and the subsequent composition of di- and tri-snRNPs (Figure 6).

#### The LSM2-8 Complex in Plants

Perea-Resa et al. (2012) reported for the first time the existence of a full functional LSM2-8 complex in plants. Arabidopsis has a LSM2-8 ring with identical structure to the one reported in yeast and metazoans and, as expected, the assembly of this complex in the nucleus is directed by the LSM8 protein (Perea-Resa et al., 2012). Experimental evidence demonstrate that the LSM nuclear complex also ensures the correct levels of U6 snRNA by promoting its stability (Perea-Resa et al., 2012; Golisz et al., 2013). Moreover, LSM8 co-immunoprecipitates with the Arabidopsis homologues of La/Lhp1 and Prp24, confirming that the LSM nuclear complex is part of a canonical U6 snRNP in plants (Golisz et al., 2013). The characterization of Arabidopsis *lsm4* and *lsm5/sad1* mutants suggested that LSM4 and LSM5 proteins are likely implicated in the control of pre-mRNA splicing (Xiong et al., 2001; Zhang et al., 2011; Cui et al., 2014). Nevertheless, as already mentioned, the fact that these proteins belong to both LSM1-7 and LSM2-8 complexes, hinders the possibility of attributing that function to one or another complex. High-coverage RNA-seq analysis using null *lsm8* mutants conclusively demonstrated that the LSM nuclear complex participates in the control of both constitutive and AS of a number of pre-mRNAs (Carrasco-López et al., 2017). This unexpected ability of the LSM2-8 complex to control the splicing of just a discrete number of pre-mRNAs indicates that the core components of eukaryotic spliceosome contribute, together with the associated proteins, to determining the spliceosome activity specificity. Moreover, this role is essential to establish the adequate gene-expression landscape in Arabidopsis (Perea-Resa et al., 2012; Golisz et al., 2013). Interestingly, several pre-mRNA targets of the LSM2-8 complex correspond to development-related genes (Perea-Resa et al., 2012; Golisz et al., 2013), suggesting that it is involved in plant development. Indeed, it was demonstrated that the LSM2-8 complex regulates different developmental processes (Perea-Resa et al., 2012). A recent study described that LSM4 and LSM5 control the splicing pattern of pre-mRNAs corresponding to important components of the clock and set the adequate length of the circadian period (Perez-Santángelo et al., 2014), which indicates that the LSM2-8 complex might also regulate the circadian rhythm in plants. Again, the participation of LSM4 and LSM5 in both LSM complexes hampers to clearly discerning the involvement of each complex in this regulation.

#### The LSM2-8 Complex in Plant Response to Abiotic Stress

**FIGURE 7:**
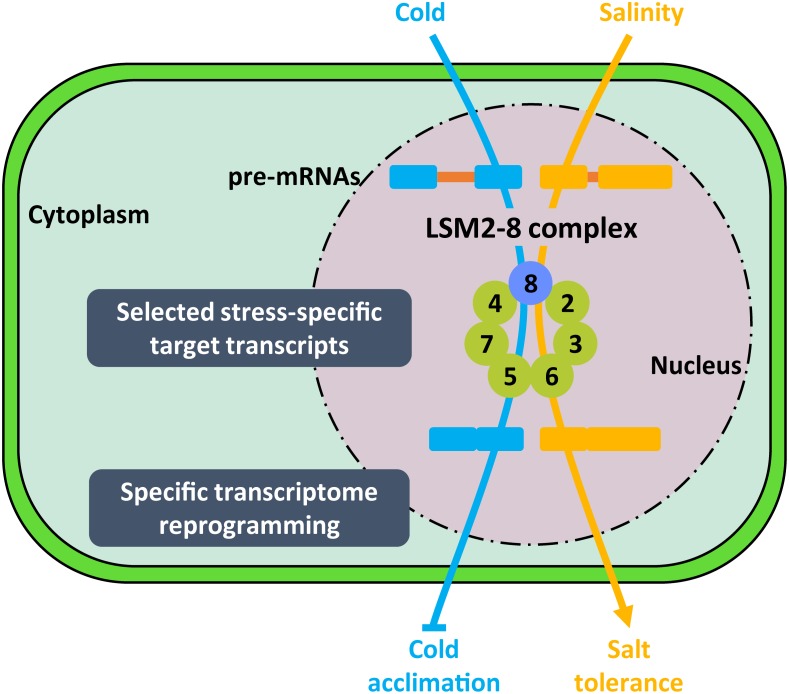
Proposed model for the function of LSM2-8 complex in plant response to abiotic stresses. Depending on the stress condition (i.e., low temperature or high salt), the LSM2-8 complex would target stress-specific transcripts encoding important regulators of plant tolerance to the corresponding condition to ensure their correct and specific pattern of splicing. The activity of the LSM2-8 complex, therefore, would shape the transcriptome reprogramming required for plant adaptation to a particular abiotic stress situation. Arrowheads and end lines indicate positive and negative regulation, respectively.

Arabidopsis *LSM8* transcripts and the corresponding protein accumulate in response to low temperature (Carrasco-López et al., 2017), which suggest that the LSM nuclear complex might also have a role in plant response to abiotic stress. On the other hand, we have already mentioned that LSM4 and LSM5 positively regulate Arabidopsis tolerance to salt stress (Xiong et al., 2001; Zhang et al., 2011; Cui et al., 2014), but if this function is carried out throughout the LSM2-8 complex remains uncertain. Concluding experimental evidence on the implication of the Arabidopsis LSM2-8 complex in abiotic stress response has been recently attained by functionally characterizing *lsm8* null mutant plants. These analyses revealed that the LSM nuclear complex differentially regulates Arabidopsis tolerance to abiotic stress. It functions as a negative regulator of the cold acclimation process, while positively controlling tolerance to salt stress (Carrasco-López et al., 2017). Deep RNA-seq experiments using *lsm8* mutant plants subjected to cold and salt stresses unveiled that the LSM2-8 complex operates by ensuring the efficiency and accuracy of the splicing of selected pre-mRNAs, depending on the adverse environmental conditions. Thus, under low temperature conditions, the complex ensures the correct splicing of a selected subset of pre-mRNAs enriched in cold-related genes, such as *MYB96*, *PRR5* or *RVE1*. In contrast, in response to high salt it guarantees the adequate splicing of a different group of pre-mRNAs, which is enriched in salt stress-related genes, such as *SAT32*, *NHX1* or *SIS* (Figure 7). In both cases, moreover, the pre-mRNAs are distinct to those whose splicing is controlled by the complex under standard conditions (Carrasco-López et al., 2017). It is worth mentioning that miss-splicing of most LSM2-8 targeted pre-mRNAs leads to the generation of NMD signatures, indicating that the complex also warrants correct levels of the corresponding functional transcripts (Carrasco-López et al., 2017). Hence, the unanticipated specificity of the nuclear complex for particular pre-mRNA targets upon plant exposure to different abiotic stress conditions seems to ensure the adequate transcriptional patterns for each stress situation and, consequently, a correct adaptive response.

In *lsm8* mutants exposed to low temperature or high salt, intron retention is, by far, the most abundant detected category of altered splicing events, evidencing that the main function of the Arabidopsis LSM2-8 complex in splicing, when plants are exposed to abiotic stress, is ensuring the full processing of introns (Carrasco-López et al., 2017). An important question that arises from these results is which are the molecular determinants underlying the specificity of the complex to target the introns that are going to be spliced under distinct adverse environments. Most genes containing targeted introns are not differentially transcribed under cold or high salt conditions, which excludes the possibility that the introns selected by the LSM nuclear complex belong to genes only or highly transcribed under a particular external stimulus. Regarding the possibility that the selected introns may include particular sequence motifs, no enrichment of sequence motifs in specific introns or particular frequencies of nucleotide sequences around their 5′ and 3′ SSs, or in their branch sites, have been found either. Interestingly, however, there are significant differences in GC content and/or length between some subsets of introns specifically spliced by the complex in response to distinct stress conditions (Carrasco-López et al., 2017). It has been proposed that secondary structures at the 5′ splice site of introns disfavor its recognition by the spliceosome and, thus, would affect their correct splicing (Ding et al., 2014b). It can be predicted that different GC content and length would end up in distinct secondary structures in these regions of the transcripts. Hence, it is tempting to speculate that particular secondary structural features of the introns could determine their specific selection by the LSM2-8 complex and, thus, by the spliceosome, in response to a given abiotic stress. The characterization of the *lsm8* mutant plants, therefore, has revealed that the core components of the spliceosome, such as the LSM2-8 complex, may regulate the activity specificity of this macromolecular machinery in an environmental condition-dependent manner, which represents a novel functional capacity for those components. Remarkably, furthermore, this function constitutes a new layer of posttranscriptional regulation in response to external stimuli in eukaryotes that seems to be essential for plant adaptation to adverse surroundings.

## Future Perspectives

Research advances in recent years have significantly expanded our understanding about the function of the LSM complexes in posttranscriptional regulation of plant response to abiotic stress. It is obvious, however, that we are still far from envisaging the molecular mechanisms regulating their function and the molecular determinants of their specificity. Data suggest that the regulatory mechanisms of plant LSM complexes take place at different levels. The methylation of LSM4 promotes its function in pre-mRNA splicing and plant tolerance to salt stress (Zhang et al., 2011). On the other hand, the activity of some components of the decapping machinery in response to osmotic stress has been shown to be governed by their phosphorylation status (Xu and Chua, 2012; Soma et al., 2017). Interestingly, LSM1 and LSM8 display amino acid sequence motives characteristic of MPK targets, and coimmunoprecipitate with some components of the MPKs family (Perea-Resa et al., 2012; Golisz et al., 2013), suggesting that the function of LSM complexes could be shaped by their differential phosphorylation pattern under a particular stress condition. Determining whether the LSM complexes may be differentially methylated, phosphorylated, or undergo some other type of posttranslational modification in response to different abiotic stresses to better adapt to challenging environments represents a research line worth to be developed in the future.

One of the most remarkable features of eukaryotic LSM complexes is that they have six common proteins. Hence, LSM1 and LSM8 have to compete for LSM2-LSM7 in order to constitute their corresponding complexes. Results obtained in yeast indicate that this competition would allow a co-regulatory mechanism of nuclear and cytoplasmic RNA processing under stressful situations (Spiller et al., 2007; Luhtala and Parker, 2009). Exploring whether this mechanism exists in plants, and if it has a role in modulating mRNA decapping or pre-mRNA splicing depending on the abiotic stress conditions is another exciting line of investigation that requires to be developed.

Another intriguing issue that needs to be approached in the future is the identity of the molecular determinants controlling the specificity of the LSM complexes to select their RNA targets in response to different adverse environmental conditions. It is well known that the levels of epigenetic marks in the chromatin influence pre-mRNA splicing in yeast and metazoans (Luco et al., 2011). In Arabidopsis, a link between the levels of the epigenetic mark histone H3 lysine 36 tri-methylation (H3K36me3) and pre-mRNA splicing has been established (Pajoro et al., 2017). Indeed, this study revealed that both, the enzymes involved in the deposition of the H3K36me3 mark and the readers of the mark, contribute to determine the patterns of AS. Considering these data and the fact that different abiotic stress conditions induce specific patterns of epigenetic marks, it is reasonable to speculate that the LSM2-8 complex could select their pre-mRNA targets in each condition depending on the particular epigenetic marks present in the chromatin of the corresponding genes. Remarkably, it has been reported that uncapped-degrading mRNAs and alternatively spliced pre-mRNAs show a no-coincident preference for some particular chemical modifications in Arabidopsis (Vandivier et al., 2015). Whether RNA chemical modifications may also significantly contribute to determine the specificity of the LSM complexes in mRNA decapping and/or pre-mRNA splicing is another interesting possibility that deserves to be explored.

## Author Contributions

All authors designed the review. CC-L, RC, and JS designed the figures. RC and JS wrote the manuscript. All authors read and approved the final version of the manuscript.

## Conflict of Interest Statement

The authors declare that the research was conducted in the absence of any commercial or financial relationships that could be construed as a potential conflict of interest.
